# Predictors of the accuracy of pulse-contour cardiac index and suggestion of a calibration-index: a prospective evaluation and validation study

**DOI:** 10.1186/s12871-015-0024-x

**Published:** 2015-04-02

**Authors:** Wolfgang Huber, Jonas Koenig, Sebastian Mair, Tibor Schuster, Bernd Saugel, Florian Eyer, Veit Phillip, Caroline Schultheiss, Philipp Thies, Ulrich Mayr, Henrik Einwächter, Matthias Treiber, Josef Hoellthaler, Roland M Schmid

**Affiliations:** 1 II. Medizinische Klinik und Poliklinik, Klinikum rechts der Isar, Technische Universität München, Ismaninger Strasse 22, D-81675 Munich, Germany; 2 Institut für Medizinische Statistik und Epidemiologie, Klinikum Rechts der Isar; Technische Universität München, Ismaninger Strasse 22, D-81675 Munich, Germany; 3 Toxikologische Abteilung, II. Medizinische Klinik und Poliklinik, Klinikum rechts der Isar, Technische Universität München, Ismaninger Strasse 22, D-81675 Munich, Germany

**Keywords:** Hemodynamic monitoring, Pulse contour analysis, Cardiac output, Cardiac index, Calibration, Transpulmonary thermodilution, Accuracy, Precision

## Abstract

**Background:**

Cardiac Index (CI) is a key-parameter of hemodynamic monitoring. Indicator-dilution is considered as gold standard and can be obtained by pulmonary arterial catheter or transpulmonary thermodilution (TPTD; CItd). Furthermore, CI can be estimated by *Pulse-Contour-Analysis (PCA)* using arterial wave-form analysis (CIpc). Obviously, adjustment of CIpc to CItd initially improves the accuracy of CIpc. Despite uncertainty after which time accuracy of CIpc might be inappropriate, recalibration by TPTD is suggested after a maximum of 8 h.

We hypothesized that accuracy of CIpc might not only depend on time to last TPTD, but also on changes of the arterial wave curve detectable by PCA itself. Therefore, we tried to prospectively characterize predictors of accuracy and precision of CIpc (primary outcome). In addition to “time to last TPTD” we evaluated potential predictors detectable solely by pulse-contour-analysis.

Finally, the study aimed to develop a pulse-contour-derived “calibration-index” suggesting recalibration and to validate these results in an independent collective.

**Methods:**

In 28 intensive-care-patients with PiCCO-monitoring (Pulsion Medical-Systems, Germany) 56 datasets were recorded. CIpc-values at baseline and after intervals of 1 h, 2 h, 4 h, 6 h and 8 h were compared to CItd derived from immediately subsequent TPTD. Results from this evaluation-collective were validated in an independent validation-collective (49 patients, 67 datasets).

**Results:**

Mean bias values CItd-CIpc after different intervals ranged between -0.248 and 0.112 L/min/m^2^. Percentage-error after different intervals to last TPTD ranged between 18.6% (evaluation, 2 h-interval) and 40.3% (validation, 6 h-interval). In the merged data, percentage-error was below 30% after 1 h, 2 h, 4 h and 8 h, and exceeded 30% only after 6 h. “Time to last calibration” was neither associated to accuracy nor to precision of CIpc in any uni- or multivariate analysis.

By contrast, the height of CIpc and particularly changes in CIpc compared to last thermodilution-derived CItd(base) univariately and independently predicted the bias CItd-CIpc in both collectives.

Relative changes of CIpc compared to CItd(base) exceeding thresholds derived from the *evaluation-collective* (-11.6% < CIpc-CItd(base)/CItd(base) < 7.4%) were confirmed as significant predictors of a bias |CItd-CIpc| ≥ 20% in the *validation-collective*.

**Conclusion:**

Recalibration triggered by changes of CIpc compared to CItd(base) derived from last calibration should be preferred to fixed intervals.

## Background

Cardiac index (CI) is a key-parameter of hemodynamic monitoring. Indicator-dilution is considered as gold standard and can be obtained by pulmonary arterial catheter or transpulmonary thermodilution (TPTD; CItd) [1-4].

Furthermore, CI can be estimated by *Pulse-Contour-Analysis (PCA)* using arterial wave-form analysis to derive stroke volume. Pulse-Contour-Analysis has been introduced in the early 1970s [5,6] and can be used for “beat by beat”-tracking of CI after initial calibration with indicator dilution or other techniques [7-13]. Finally, Pulse-Contour-Analysis combined with biometric and empirical data can provide an uncalibrated estimate of CI [14-18].

There is consensus that indicator dilution techniques provide best accuracy. However, due to rapid and unpredictable changes in hemodynamics in critically ill, the usefulness of *intermittent* CI-determinations has been questioned, and *continuous* CI-monitoring might enhance sensitivity of CI-monitoring [19].

Although numerous studies have demonstrated appropriate correlation of PCA-derived CI (CIpc) immediately after calibration [7-13], the need for recalibration and the interval to the next calibration still are matter of debate [20-24]. As a minimum consensus, most manufacturers recommend recalibration after a maximum of 8 h.

However, some data suggest more frequent recalibration with intervals as short as one hour [20,21]. Nevertheless, there are a number of reasons to limit the frequency of TPTDs:

TPTD requires a certain amount of time of qualified personal.

Furthermore, calibration with a limited number of TPTDs carries a certain risk of imprecision that might sum up in case of repeated measurements. At least three TPTDs are required to provide acceptable precision ≤10% and detection of changes in CI ≥15% that are generally considered as clinically relevant [25-27].

However, repeated TPTDs with at least 45 ml per triplicate measurement might result in a substantial fluid load with impact on hemodynamics itself.

With the data available being not fully consistent and in part retrospective, there is a lack of studies prospectively evaluating the impact of pre-defined periods without calibration and other possible predictors of imprecision, all systematically determined within the same patient.

We hypothesized that accuracy of CIpc might not only depend on time to last TPTD, but also on changes of the arterial wave curve detectable by PCA itself.

Therefore, the aims of our study were

- to prospectively investigate accuracy and precision of CIpc after pre-defined intervals of 1 h, 2 h, 4 h, 6 h and 8 h after the last TPTD,
- to evaluate the impact of “time to the last TPTD-calibration” and other factors on the agreement of CIpc and CItd,
- to derive a “calibration index” solely from PCA- parameters comprising a formula predicting the disagreement of CIpc and CItd and an alarming-function suggesting recalibration when predicted disagreement exceeds user-defined limits (e.g. >15% or >0.5 L/min/m^2^), and
- to validate these results in an independent second collective.

## Methods

The study was approved by the institutional review board (Ethikkommission der Fakultät für Medizin der Technischen Universität München; Ismaninger Straße 22; 81675 München). The need of informed consent was waived.

In 28 consecutive patients (evaluation collective) with PiCCO-monitoring treated in a general intensive care unit (ICU) or a toxicology ICU, 56 data-sets each including a total of 6 triplicate TPTDs at baseline and after intervals of 1 h, 2 h, 4 h, 6 h and 8 h after the last TPTD were recorded within 21 hours. Since follow-up TPTDs re-calibrated CItd, these measurements also provided the baseline CItd for the next interval. The sequence of intervals was randomized.

Results derived from this *evaluation-collective* were validated in an independent *validation-collective* of 49 patients with 67 datasets. Due to practical reasons (e.g. transport, external intervention) 21/615 (3%) of measurements could not be performed within ±10 min of the scheduled time and could not be included in the final analysis. A total of 123 datasets with 594 measurements were finally analyzed.

CIpc and CItd were determined using the PiCCO-System (Pulsion Medical Systems, Munich, Germany) as described before [21,28]. Briefly, a 5-French thermistor-tipped arterial line (Pulsiocath, Pulsion Medical Systems) placed in the femoral artery and a hemodynamic monitor (PiCCO-Plus; PiCCO-2, Pulsion Medical Systems) were used for analysis of pulse-contour and a thermodilution curve after injection of a cold indicator-bolus (15 mL saline 0.9%) through a central-venous catheter (CVC).

CIpc recorded immediately before recalibration with triplicate TPTD was compared to CItd derived from the new TPTD.

Primary endpoint: Analysis of parameters independently associated with the bias CItd-CIpc. These parameters included “time to last calibration” as well as factors continuously provided by pulse contour analysis and their changes compared to baseline.

Secondary endpoints: Analysis of parameters associated with bias CItd-CIpc exceeding pre-defined thresholds (20%, 15% of CItd and 0.5 L/min/m^2^) and development of a “calibration-index” suggesting recalibration based on parameters derived from pulse-contour-analysis and/or last thermodilution.

### Statistics

To describe accuracy and precision of CIpc compared to CItd after different intervals, we performed analyses according to Bland-Altman [29]. To avoid analysis of repeated measurements and different numbers of measurements, Bland-Altman-analyses included only one dataset per patient (first series) and were performed separately for each interval. Percentage-error was calculated as described previously [30].

All other analyses were performed including all datasets except as indicated. For appropriate consideration of multiple measurements per patient in these analyses, uni- and multivariable regression models were fitted in a “Generalized Linear Mixed Model” (GLMM) framework. ROC-analyses were performed to assess discriminative ability of predictor variables regarding pre-defined thresholds of the bias CItd-CIpc (exceeding 20%, 15% of CItd or 0.5 L/min/m^2^). Percentages were calculated based on the measurements with valid data. In the course of GLMM-analysis, standard-errors of regression coefficients were reported. In order to consider repeated measurements per individual, partial correlation-coefficients (r_part_) were calculated for bivariate correlation.

Predictors of bias exceeding critical thresholds derived from the evaluation-collective were analysed in the validation-collective based on ROC- and percentage-error-analysis.

All statistical analyses were performed by statistician co-author TS using IBM SPSS Statistics 21 (SPSS Inc., Chicago, IL, USA).

## Results

### Patients characteristics and interventions

Patients characteristics are demonstrated in Table 1.

**Table 1:** **Patients characteristics and interventions**

	Evaluation	Validation	Merged data
**Patients characteristics**			
Number of patients	28	49	77
Male	19 (67.9%)	20 (40.8%)	39 (50.6%)
Female	9 (32.1%)	29 (59.2%)	38 (49.4%)
Height [m]	1.73 ± 0.076	1.68 ± 0.077	1.70 ± 0.083
Weight [kg]	75.4 ± 14.1	70.21 ± 17.2	72.2 ± 16.2
Age [years]	60.2 ± 11.8	61.1 ± 15.0	60.7 ± 13.9
APACHE-II	23.5 ± 5.6	22.3 ± 8.6	22.7 ± 7.6
**Etiology**			
ARDS	8/28 (28.6%)	17/49 (34.7%)	25/77 (32.5%)
Liver Disease	8 /28(28.6%)	10/49 (20.4%)	18/77 (23.4%)
Gastric Disease	3/28 (10.7%)	3/49 (6.1%)	6/77 (7.8%)
Sepsis	3/28 (10.7%)	11/49 (22.4%)	14/77 (18.2%)
Cardiogenic Shock	3/28 (10.7%)	4/49 (8.2%)	7/77 (9.1%)
Affection of the central nervous system	3/28 (10.7%)	4/49 (8.2%)	7/77 (9.1%)
**Series of Measurements including**			
Mechanical ventilation	39/55 (70.9%)	33/67 (49.3%)	72/122 (59.0%)
Use of catecholamines	30/56 (53.6%)	31/67 (46.3%)	61/123 (49.6%)
**Series of Measurements with Interventions**			
Change in catecholamine dose	29/56 (51.8%)	29/67 (43.3%)	58/123 (47.2%)
Terlipressin	4/56 (7.1%)	5/67 (7.5%)	9/123 (7.3%)
Change in Terlipressin	3/56 (5.4%)	3/67 (4.5%)	6/123 (4.9%)
Other vasoactive drug (Clonidin…)	10/56 (17.9%)	1/67 (1.5%)	11/123 (8.9%)
Change in other vasoactive drug	10/56 (17.9%)	1/67 (1.5%)	11/123 (8.9%)
Any vasoactive drug	38/56 (67.9%)	33/67 (49.3%)	71/123 (57.7%)
Change in any vasoactive drug	37/56 (66.1%)	31/67 (46.3%)	68/123 (55.3%)
Renal replacement therapy (RRT)	12/56 (21.4%)	7/67 (10.4%)	19/123 (15.4%)
Change in RRT	11/56 (19.6%)	7/67 (10.4%)	18/123 (14.6%)
Change in positioning (prone/supine)	1/56 (1.8%)	3/67 (4.5%)	4/123 (3.3%)
Pleural puncture	1/56 (1.8%)	2/67 (3.0%)	3/123 (2.4%)
Transfusion	5/56 (8.9%)	2/67 (3.0%)	7/123 (5.7%)
Cardioversion	1/56 (1.8%)	1/67 (1.5%)	2/123 (1.6%)
Change in heart rhythm	4/56 (7.1%)	3/67 (4.5%)	7 /123 (5.7%)
Change in ventilation mode	10/56 (17.9%)	1/67 (1.5%)	11/123 (8.9%)

About 50% of all TPTD-measurements were performed during the use of catecholamines, 59% during mechanical ventilation. Within 123 series of measurements, there were changes in the use of vasoactive drugs in 71/123 (58%), in positioning (prone vs. supine) in 4/123 (3%), initiation or termination of renal replacement-therapy (RRT) in 18/123 (15%) and some kind of other intervention (volume challenge, cardiopulmonary resuscitation, endoscopy, tracheotomy) in 31/123 (25%) datasets.

### Mean bias of CItd and CIpc

Neither in the evaluation-collective (4.15 ± 1.46 vs. 4.09 ± 1.41 L/min/m^2^; p = 0.265) nor in the validation-collective (4.07 ± 1.27 vs. 4.07 ± 1.21 L/min/m^2^; p = 0.555) there was a significant difference between CIpc and CItd. Mean bias values were -0.0606 ± 0.603 and 0.00261 ± 0.610 L/min/m^2^, respectively.

### Bias values exceeding pre-defined thresholds

Despite low *mean* bias values, “CItd-CIpc” exceeded critical thresholds in a relevant number of *single comparisons* (Table 2). In the merged data, bias values exceeding ±20%, ±15% and ±0.5 L/min/m^2^ were observed in 85/594 (14.3%), 138/594 (23.2%) and 166/594 (27.9%) of measurements.

**Table 2:** Percentages of bias (CItd-CIpc)*-values exceeding critical thresholds

Threshold of Bias CItd-CIpc	Evaluation	Validation	Merged
Bias CItd-CIpc ≥ 20%	13/280 (4.6%)	20/314 (6.4%)	33/594 (5.6%)
Bias CItd-CIpc ≤ 20%	25/280 (8.9%)	27/314 (8.6%)	52/594 (8.8%)
|Bias CItd-CIpc| ≥ 20%	38/280 (13.6%)	47/314 (15.0%)	85/594 (14.3%)
Bias CItd-CIpc ≥ 15%	26/280 (9.3%)	36/314 (11.5%)	62/594 (10.4%)
Bias CItd-CIpc ≤ 15%	40/280 (14.3%)	36/314 (11.5%)	76/594 (12.8%)
|Bias CItd-CIpc| ≥ 15%	66/280 (23.6%)	72/314 (22.9%)	138/594 (23.2%)
Bias CItd-CIpc ≥ 0.5 L/min/m^2^	34/280 (12.1%)	43/314 (13.7%)	77/594 (13.0%)
Bias CItd-CIpc ≤ 0.5 L/min/m^2^	46/280 (16.4%)	43/314 (13.7%)	89/594 (15.0%)
|Bias CItd-CIpc| ≥ 0.5 L/min/m^2^	80/280 (28.5%)	86/314 (27.4%)	166/594 (27.9%)

*CItd: Thermodilution-derived Cardiac Index.

*CIpc: Pulse-contour-derived Cardiac Index.

### Impact of interval to last TPTD calibration

**Figure 1:**
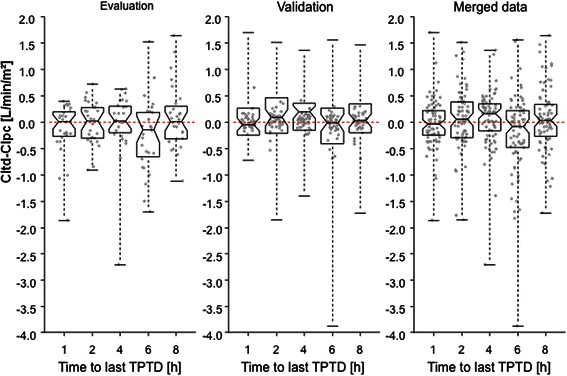
**Notched boxplots demonstrate that bias CItd-CIpc did not increase over time and did not differ after various times to last calibration.** Only if the notches of two boxplots do not overlap, this is ‘strong evidence’ that the two medians differ [31,32]. CItd: Thermodilution-derived Cardiac Index. CIpc: Pulse-contour-derived Cardiac Index. TPTD: Transpulmonary thermodilution.

Figure 1 and Table 3 demonstrate mean bias and percentage-error values of CIpc vs. CItd 1 h, 2 h, 4 h, 6 h and 8 h after the last TPTD calibration.

**Table 3:** **Percentage-error and bias CItd-CIpc values after 1 h, 2 h, 4 h, 6 h and 8 h without thermodilution (one data set per patient)**

	Evaluation		Validation		Merged data	
Interval to last thermodilution	Percentage Error	Mean bias [L/min/m^2^]	Percentage Error	Mean bias [L/min/m^2^]	Percentage Error	Mean bias [L/min/m^2^]
1 h	23.2%	−0,144 ± 0,501	23.4%	0.026 ± 0.465	23.5	−0.039 ± 0.483
2 h	18.6%	−0,021 ± 0,400	32.5%	0.112 ± 0.660	27.7	0.061 ± 0.576
4 h	28.5%	−0,020 ± 0,618	24.4%	0.097 ± 0.490	26.1	0.054 ± 0.539
6 h	30.9%	−0,248 ± 0,690	40.3%	−0.200 ± 0.861	36.6	−0.218 ± 0.794
8 h	27.9%	0,082 ± 0,600	31.0	0.033 ± 0.632	29.6	0.062 ± 0.615

Mean bias values in general were low and ranged between -0.248 (evaluation-collective, after 6 h-interval) and 0.112 L/min/m^2^ (validation-collective after 2 h-interval). In both collectives mean bias values were not dependent on time to last calibration (Table 3, Figure 1). Notched boxplots further support that bias CItd-CIpc did not increase over time and did not differ after various times to last calibration: Only if the notches of two boxplots do not overlap, the two medians differ [31,32].

Percentage-error values ranged between 18.6% (evaluation, 2 h-interval) and 40.3% (validation, 6 h-interval; Table 3).

**Figure 2:**
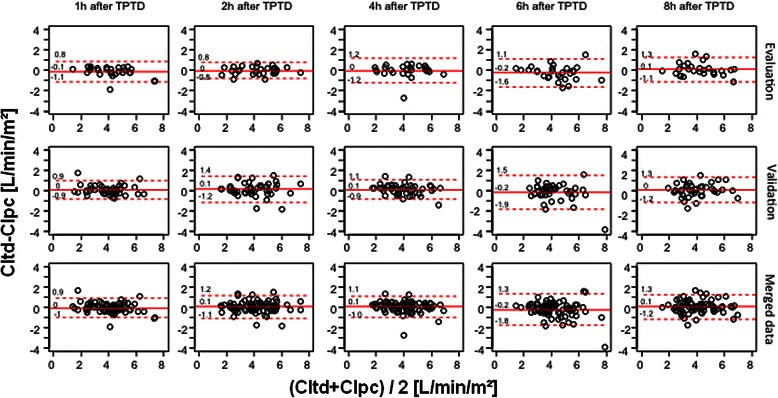
**Bland-Altman plots performed separately for each interval and including only one data set per patient demonstrate low bias CItd-CIpc (solid line) and comparable limits of agreement (dashed lines) 1 h, 2 h, 4 h, 6 h and 8 h after the last thermodilution.** CItd: Thermodilution-derived Cardiac Index. CIpc: Pulse-contour-derived Cardiac Index.

Bland-Altman-diagrams (one data set per patient; Figure 2) with lower and upper limits of agreement and bias-values demonstrate comparable accuracy and precision for the different intervals to last TPTD.

### Univariate analysis of potential predictors of bias CItd-CIpc including “time to last TPTD”

**Figure 3:**
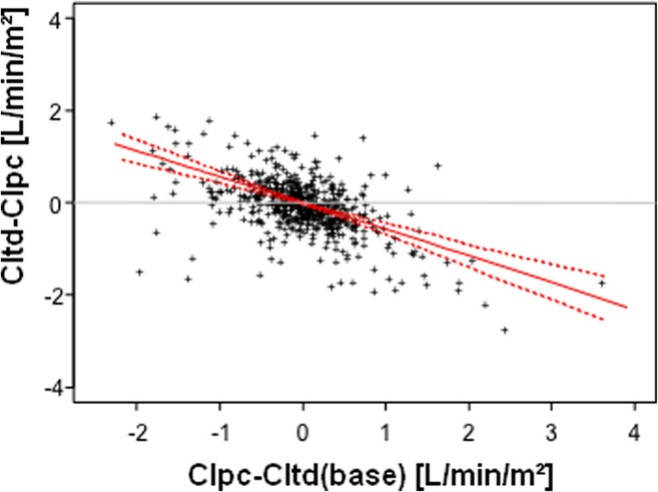
**CItd-CIpc was significantly associated to the difference CIpc-CItd(base): r**_**part**_ **= -0.606; p < 0.001 (merged data).** CItd: Thermodilution-derived Cardiac Index. CIpc: Pulse-contour-derived Cardiac Index. CItd(base): Cardiac index measured at previous (baseline) thermodilution. Dashed lines represent the limits of the 95%-bootstrap-confidence-intervals for the regression line (solid line).

**Figure 4:**
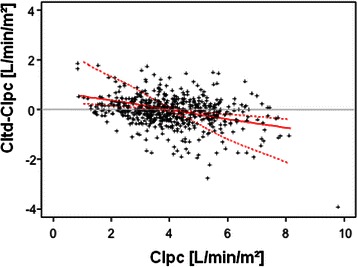
**CItd-CIpc was also significantly associated to CIpc itself: r**_**part**_ **= -0.466; p < 0.001 (merged data).** CItd: Thermodilution-derived Cardiac Index. CIpc: Pulse-contour-derived Cardiac Index. CItd(base): Cardiac index measured at previous (baseline) thermodilution. Dashed lines represent the limits of the 95%-bootstrap-confidence-intervals for the regression line (solid line).

Similarly to the data based on one data set per patient (Figures 1 and 2 and Table 3), the interval to last TPTD was not associated to the bias CItd-CIpc when including repeated data sets for correlation analysis. Comparison of bias CItd-CIpc to “time to last calibration” provided poor coefficients of partial correlation r_part_ and p-values in evaluation-collective (r_part_ = -0.09; p = 0.536), validation-collective (r_part_ =0.083; p = 0.605) and merged data (r_part_ = 0.076; p = 0.363). As demonstrated in Table 4 and Figure 3, bias CItd-CIpc was most strongly associated to the difference CIpc-CItd(base) (r_part_ = -0.592 (evaluation-collective), r_part_ = -0.630 (validation-collective) and r_part_ = -0.606 (merged data); p < 0.001 for both collectives and merged data). The second strongest predictor of the bias CItd-CIpc was CIpc itself (r_part_ = -0.367 (evaluation), r_part_ = -0.573 (validation) and r_part_ = -0.466 (merged data; p < 0.001 for both collectives and merged data; Table 4; Figure 4).

**Table 4:** Partial correlation of predictors of absolute and relative bias (relative bias: CItd-CIpc/CItd)* (all data)

Parameter	Correlation to “absolute bias” (coefficient of partial correlation)			Correlation to “relative bias” (coefficient of partial correlation)		
Collective	Evaluation	Validation	Merged	Evaluation	Validation	Merged
Intervall	−0.090 (p = 0.536)	0.083 (p = 0.605)	0.076 (p = 0.363)	−0.014 (p = 0.997)	0.083 (p = 0.597)	0.039 (p = 0.836)
CIpc	−0.367 (p < 0.001)	−0.573 (p < 0.001)	−0.466 (p < 0.001)	−0.355 (p < 0.001)	−0.529 (p < 0.001)	−0.422 (p < 0.001)
CIpc-CItd(base) [abs]^†^	−0.592 (p < 0.001)	−0.630 (p < 0.001)	−0.606 (p < 0.001)	−0.529 (p < 0.001)	−0.577 (p < 0.001)	−0.547 (p < 0.001)
CIpc-CItd(base) [rel]	−0.564 (p < 0.001)	−0.632 (p < 0.001)	−0.574 (p < 0.001)	−0.570 (p < 0.001)	−0.612 (p < 0.001)	−0.583 (p < 0.001)
Delta-dPmax [abs]^‡^	−0.279 (p < 0.001)	−0.115 (p = 0.325)	−0.170 (p = 0.001)	−0.240 (p = 0.001)	−0.136 (p = 0.173)	−0.168 (p = 0.001)
Delta-dPmax [rel]	−0.286 (p < 0.001)	−0.091 (p = 0.533)	−0.162 (p = 0.002)	−0.255 (p < 0.001)	−0.110 (p = 0.353)	−0.166 (p = 0.002)
Delta-MAP [abs]	−0.257 (p < 0.001)	−0.114 (p = 0.324)	−0.179 (p = 0.001)	−0.224 (p = 0.003)	−0.137 (p = 0.164)	−0.180 (p < 0.001)
Delta-MAP [rel]	−0.243 (p = 0.001)	−0.119 (p = 0.286)	−0.165 (p = 0.002)	−0.196 (p = 0.015)	−0.157 (p = 0.080)	−0.162 (p = 0.002)
Delta-PP [abs]^§^	−0.356 (p < 0.001)	−0.188 (p = 0.023)	−0.260 (p < 0.001)	−0.323 (p < 0.001)	−0.222 (p = 0.004)	−0.262 (p < 0.001)
Delta-PP [rel]	−0.336 (p < 0.001)	−0.179 (p = 0.036)	−0.239 (p < 0.001)	−0.309 (p < 0.001)	−0.202 (p = 0.011)	−0.243 (p < 0.001)
Delta-RRsyst [abs]	−0.321 (p < 0.001)	−0.155 (p = 0.093)	−0.227 (p < 0.001)	−0.284 (p < 0.001)	−0.205 (p = 0.009)	−0.233 (p < 0.001)
Delta-RRsyst [rel]	−0.320 (p < 0.001)	−0.151 (p = 0.106)	−0.223 (p < 0.001)	−0.278 (p < 0.001)	−0.197 (p = 0.013)	−0.226 (p < 0.001)
Delta-RRdiast [abs]	−0.148 (p = 0.116)	−0.085 (p = 0.587)	−0.090 (p = 0.214)	−0.125 (p = 0.238)	−0.096 (p = 0.476)	−0.096 (p = 0.162)
Delta-RRdiast [rel]	−0.182 (p = 0.029)	−0.089 (p = 0.549)	−0.114 (p = 0.068)	−0.173 (p = 0.044)	−0.102 (p = 0.420)	−0.118 (p = 0.051)

^*^CItd: Thermodilution-derived Cardiac Index; CIpc: Pulse-contour-derived Cardiac Index.

^†^CIpc-CItd(base): Cardiac index measured at previous (baseline) thermodilution.

^‡^dPmax: “Index of Left Ventricular Contractility”.

^§^PP: Pulse pressure.

[rel]: relative changes compared to time of the last thermodilution.

[abs]: absolute changes compared to time of the last thermodilution.

Among the other predictors, a modest association to the bias CItd-CIpc was found for changes in Pulse Pressure (PP) which was significant in evaluation- (r = -0.356; p < 0.001), validation- (r = -0.188; p = 0.023) and merged collective (r = -0.260; p < 0.001).

In addition to these associations of *absolute* bias CItd-CIpc to *absolute* changes in the above-mentioned predictors, *relative* bias (CItd-CIpc)/CItd was similarly associated to *relative* changes in the predictors (Table 4).

### Multivariate analysis regarding prediction of absolute bias CItd-CIpc

In multivariate GLMM-analysis, absolute bias CItd-CIpc was independently associated to CIpc-CItd(base) (p < 0.001) and to CIpc itself (p < 0.001), but not the interval to the last TPTD. These findings were consistent for evaluation, validation and merged data. Similar results were obtained for “relative bias” CItd-CIpc/CItd (data not shown).

### Univariate ROC-analysis regarding critical thresholds of bias CItd-CIpc

**Figure 5:**
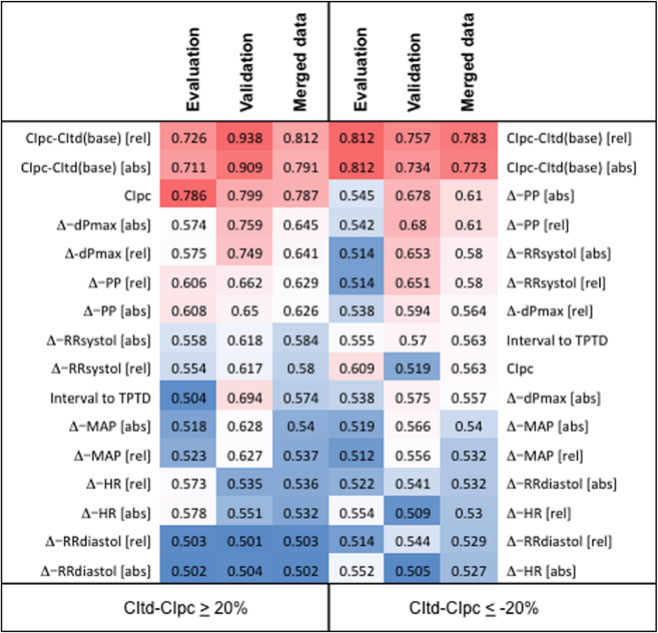
**Thermoplot demonstrating “Area under the Curve” (AUC) values from Receiver Operating Characteristic (ROC)-analyses regarding bias CIt-CIpc exceeding ≥20% of CItd.** Y-axis ranges from 0.5 (worthless for prediction; intense blue) to 1.0 (best prediction; intense red). The variables were aligned according to merged data. CItd: Thermodilution-derived Cardiac Index. CIpc: Pulse-contour-derived Cardiac Index. CItd(base): Cardiac index measured at previous (baseline) thermodilution. dPmax: “Index of Left Ventricular Contractility”. PP: Pulse Pressure. TPTD: Transpulmonary thermodilution. MAP: mean arterial pressure. RRsyst, RRdiast: systolic and diastolic pressure. HR: Heart rate. Δ-values compare data to corresponding values at the time of the last TPTD. [rel]: relative changes compared to time of the last TPTD. [abs]: absolute changes compared to time of the last TPTD.

Bias values exceeding ±15%, ±20% and ±0.5 L/min/m^2^ in general were best predicted by absolute or relative changes in CIpc compared to CItd derived from the previous TPTD (CIpc-CItd(base)). Figure 5 (thermoplot) demonstrates ROC-AUCs regarding bias values exceeding ±20%.

E.g. a decrease in CIpc-CItd(base) of at least 11.62% provided a sensitivity, specificity and accuracy of 85%, 89% and 89% to predict a bias CItd-CIpc ≥20% in the evaluation-collective. An increase of at least 7.43% in CIpc-CItd(base) provided a sensitivity, specificity and accuracy of 76%, 76% and 76% to predict a bias CItd-CIpc ≤ -20%.

### Multivariate ROC-analysis regarding critical thresholds of bias CItd-CIpc

Multivariate analysis demonstrated independent association of relative changes in CIpc-CItd(base) to relative bias (CItd-CIpc)/CItd exceeding ±15%, 20% and 0.5 L/min/m^2^. By contrast, “interval to last TPTD” was not independently associated to relative bias (CItd-CIpc)/CItd exceeding these thresholds.

**Figure 6:**
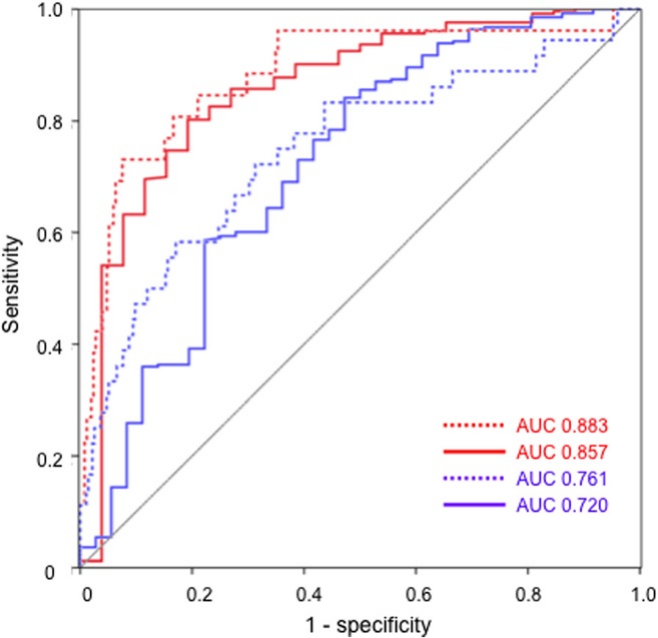
**Receiver Operating Characteristic (ROC)-analyses regarding bias CIt-CIpc exceeding ≥15%.** Solid lines represent predictuion by CIpc-CItd(base), dashed lines are derived from a multivariate model including changes in CIpc-CItd(base) and in dPmax in the evaluation-collective. Red lines: Evaluation-collective. Blue lines: Validation-collective. CItd: Thermodilution-derived Cardiac Index. CIpc: Pulse-contour-derived Cardiac Index. CItd(base): Cardiac index measured at previous (baseline) thermodilution.

Predictive capabilities of relative changes in “CIpc-CItd(base)” regarding several thresholds could be further improved by also including changes in “Index of Left Ventricular Contractility” (dPmax) (Figure 6) or changes in PP in a GLMM-derived multivariate model.

E.g. a model derived from the evaluation-collective including changes in CIpc-CItd(base) and in dPmax slightly improved ROC-AUC regarding relative bias exceeding ≥15% in the evaluation-collective (AUC 0.883 vs. 0.857) as well as in the validation-collective (0.761 vs. 0.720) (Figure 6).

### Validation of predictors of substantial bias derived from the evaluation collective in the validation collective

Analysis of evaluation data demonstrated that a decrease in CIpc-CItd(base) of at least 11.62% or an increase of at least 7.43% significantly predicted a bias CItd-CIpc exceeding ±20% (see above).

To assess the potential practical use of these thresholds derived from the *evaluation*-collective in the *validation-*collective, we compared the 8 h percentage-error of patients in the *validation*-collective staying within and without these critical thresholds. In case of repeated inclusion, only the first 8 h-observation- period was analysed. 8 h-measurements in 26 validation-patients staying within the critical evaluation-thresholds of relative changes in CIpc compared to CItd(base) (-11.62% < (CIpc-CItd(base)/CItd(base) < 7.43%) provided a percentage-error of 22.6% compared to 44.0% for those 16 validation-patients outside of these evaluation-thresholds. Similar analysis of the validation-data after a 1 h-interval demonstrated a percentage-error of 9.7% in 29 patients staying within the critical thresholds of relative changes in CIpc compared to CItd(base) compared to 36.7% for patients outside of these thresholds.

### Suggestion for a calibration-index-formula

To provide a first suggestion for a future calibration-index, we finally performed multiple regression analysis of the merged data resulting in a formula predicting bias CItd-CIpc:

$$ \mathrm{Estimated}\ \mathrm{absolute}\ \mathrm{bias}:\ 0.208 - 0.528*\Big(\mathrm{CIpc}-\mathrm{CItd}\left(\mathrm{base}\right) - 0.052*\mathrm{CIpc}. $$

CIpc (p = 0.003) and CIpc-CItd(base) (p < 0.001) were independently associated to CItd-CIp, whereas the other parameters included in the GLMM-analysis (time to last calibration, changes in PP and in dPmax) failed sig0nificance.

## Discussion

Repeated calibration of Pulse-Contour-Analysis-derived CIpc by thermodilution is considered to improve accuracy and precision of CIpc [3-13,20-27,31]. However, frequent recalibration is time-consuming, might result in inaccuracies itself and in a substantial fluid-load. Current suggestion to repeat calibration after a maximum of 8 h is in part based on a lack of studies investigating longer calibration-free periods. Several studies suggest more frequent TPTD up to once per hour [20,21]. However, “time to last calibration” so far has not been independently associated with the bias CIpc-CItd in a prospective study.

Therefore, we prospectively evaluated the accuracy of CIpc after pre-defined calibration-free periods. The main results of this study can be summarized as follows:

Mean bias of CIpc vs. CItd was acceptable after all time-periods. However, about 23% of CIpc-values deviated ≥15% from immediately subsequent CItd. This emphasizes the need for repeated recalibration.

Adaptation of recalibration to a fixed time-based scheme is not substantiated by our data, since “time to last calibration” was neither associated to accuracy nor precision of CIpc.

By contrast, relative and absolute changes in CIpc compared to the last TPTD-derived CItd(base) were independent predictors of relative and absolute bias CItd-CIpc.

Critical thresholds of changes in CIpc compared to CItd(base) derived from the *evaluation-collective* were confirmed as predictors of the bias CItd-CIpc in the *validation-collective*.

Multivariate analyses suggest that more complex mathematical models also including CIpc itself, changes in PP and dPmax might further improve prediction of disagreement between CIpc and CItd.

In our study CIpc provided appropriate accuracy irrespective of the interval to last TPTD with mean bias values between -0.21and 0.068 L/min/m^2^ (merged data). These low mean bias values in all subgroups are in accordance with two studies evaluating PiCCO-derived CIpc with mean bias values of 0.03-0.16 L/min/m^2^ [20] and 0.06-0.29 L/min/m^2^ [24] after different intervals to last TPTD [20,24] and different dosages of noradrenalin [24].

Most of the other previous studies similarly demonstrated appropriate *accuracy* of PiCCO-derived CIpc [3-13,21-24]. However, data on *precision* of CIpc are more conflicting. This might be in part related to the setting of the studies: While early studies were aimed at “feasibility” of CIpc and global percentage-error evaluation in selected collectives [3-9], more recent trials analysed more heterogeneous populations also allowing subgroup-analyses regarding precision [20,24].

Driven by the practical need to define “when” to re-calibrate, *time-dependency* of CIpc-accuracy is an obvious hypothesis. However, this is neither well substantiated by previous investigations nor by our study: Although percentage-error was within the critical threshold of 30% only within the 1st hour in Hamzaoui’s study, time to last TPTD was not an independent predictor of precision in multivariate analysis [20].

In our merged data, percentage-error was below 30% within the first 4 hours and after 8 hours and exceeded 30% only after 6 hours. In addition to percentage-error comparison after different times to last TPTD, we performed univariate and multivariate analysis regarding agreement of single CIpc-values with CItd in two different collectives and in the merged data. Furthermore, we performed notched box-plot-analyses- favouring comparison of medians over means - for pre-defined intervals to last TPTD. None of these analyses provided evidence for time-dependency of the agreement of CIpc and CItd.

This is also in accordance with a study by Gruenewald et al. who did not find any hints for an association of CIpc-precision with interval to last TPTD [24].

In general, assessment of CIpc is mainly based on the assumption that left-ventricular stroke-volume is proportional to the area under the systolic portion of the arterial pressure-curve (AUSPC). Depending on compliance and systemic vascular resistance, identical AUSPC-values result in different stroke-volumes. Therefore, most of the pulse-contour-technologies try to correct for these individual factors. These individual factors can be assumed to be composed of *static* (individual biometry: age, gender, height, weight etc.) and *dynamic* components (changes in compliance and resistance/impedance).

Early pulse-contour-approaches were mainly based on *intermittent* re-adjustment. More recent approaches also tried *continuous* correction based on more sophisticated waveform-analysis also including shape of the waveform, position of the dicrotic notch and analysis of the post-systolic area behind the dicrotic notch. This part represents passive emptying of the aorta due to the Windkessel-effect [10]. Additionally, pulse-contour-algorithms include empirical and biometric data to a different extent. This finally resulted in approaches of CIpc-assessment exclusively based on pulse-contour-analysis, empiric and biometric data, thus totally rejecting any calibration [14-18].

**Figure 7:**
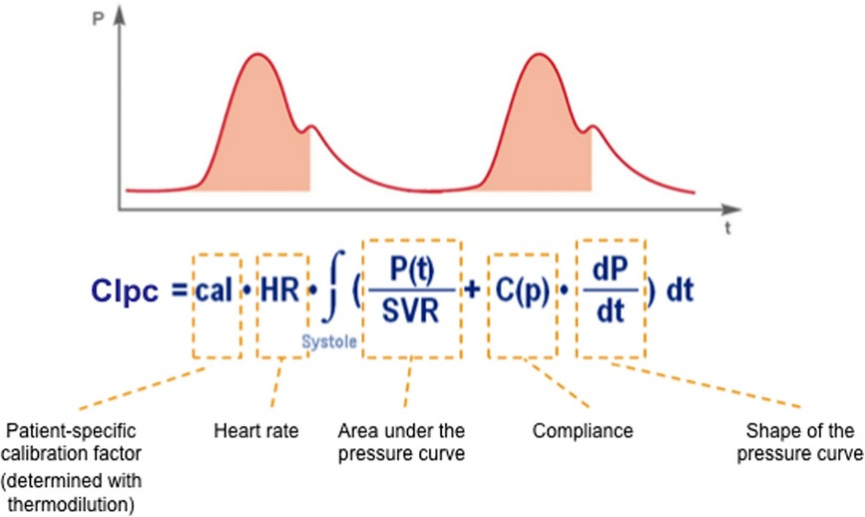
**Pulse-contour algorithm of the PiCCO-device.** CIpc: Pulse-contour-derived Cardiac Index. SVR: Systemic Vascular Resistance. P(t): arterial pressure at any time. C(p): arterial compliance continuously adjusted for arterial pressure (proprietary algorithm).

The algorithm used in recent PiCCO-devices is based on *intermittent* recalibration as well as *continuous* adjustment. With the exact algorithm being proprietary, it can be assumed that TPTD-derived calibration has impact on a “patient-specific calibration-factor” remaining constant until the next calibration (“cal”; Figure 7). Furthermore, calibration intermittently modifies continuous adjustment of Systemic Vascular Resistance (SVR) and compliance (“C(p)”).

This might in part explain that parameters adjustable by the above-mentioned formula such as changes in heart rate and arterial pressure were not substantially associated to the deviation of CIpc in our study. By contrast, changes in CIpc compared to CItd(base) and CIpc itself were independently associated with the deviation of CIpc. Independent association of CIpc to the bias CItd-CIpc suggests a systematic deviation which offers potential of systematic correction for an improved algorithm.

CIpc-CItd(base) was - by far - the most important predictor of inaccuracy of CIpc. With these changes being easily and continuously detectable by pulse-contour itself, our data suggest this parameter as a main component of a “calibration-index” triggering recalibration. Prediction of CIpc-deviation exceeding pre-defined thresholds was - in part - improved by including changes in PP and/or dPmax.

However, even a calibration-index restricted to this single predictor “CIpc-CItd(base)” provided ROC-AUCs between 0.75 and 0.81 in the prediction of CIpc-deviation exceeding several critical thresholds.

To investigate reproducibility of these data, we validated a calibration-index based on cut-offs derived from 28 evaluation-patients: PE-values were markedly lower for *validation*-patients-measurements within *evaluation*-measurements-derived thresholds (changes in CIpc compared to CItd(base) between -11.6% and +7.4%) 8 h after TPTD (22.6% vs. 44.0%), and even 1 h after TPTD (9.7% vs. 36.7%).

In addition to our findings of an association of inaccuracy of CIpc to CIpc-CItd(base) and CIpc itself, a number of clinical and/or non-hemodynamic predictors might be associated with reduced accuracy of CIpc.

Data on the impact of (changes in) vasopressor-therapy are -in part- conflicting: In an animal model, Bein and co-workers demonstrated marked deteriorated accuracy and precision after haemorrhage and application of noradrenalin [23]. In a recent study in 73 ICU-patients, the authors demonstrated *improved* accuracy of CIpc in patients with high doses of noradrenalin (>0.1 μg/kg/min) compared to lower doses of noradrenalin or no noradrenalin [24]. This might be explained by noradrenalin -induced arterial stiffness stabilizing compliance and resistance.

Furthermore, changes in SVR have been suggested as predictors of inaccuracy of CIpc. Rodig et al. demonstrated marked impaired bias and precision of CIpc after marked changes in SVR >60% induced by phenylephrine [7]. Yamashita et al. showed reduced accuracy and precision of CIpc after SVR-decreases induced by prostaglandin [33]. However, in Hamzaoui’s study neither univariate nor multivariate analysis demonstrated an association of changes in SVR to the agreement of CIpc and CItd [20].

Among several other parameters and interventions, increased IAP [22], haemorrhage [23] and volume resuscitation [22] have been associated with decreased accuracy of CIpc.

### Practical implications

The present recommendation to recalibrate after “8 h or in case of instability or events probably associated with inaccuracy of CIpc” is difficult to perform: Even under study conditions TPTD-intervals frequently exceed 8 h [24].

Regarding an increasing number of factors and interventions (see Table 1) potentially associated to inaccuracy of CIpc, permanent screening for these factors is cumbersome and hardly feasible.

Furthermore, many of the above-mentioned events associated to CIpc-inaccuracy (vasopressors, hemorrhage, increased intra-abdominal pressure etc.) can be assumed to result in changes in arterial pulse-wave. Our data support that a calibration-index derived solely and continuously from pulse-contour-analysis might be a useful tool to improve the yield of relevant TPTD-measurements and to reduce “routine”-measurements passed down from devices incapable of combining intermittent and continuous monitoring. Summarizing different analyses of this study, re-calibration should be considered in case of changes of CIpc of more than 10% compared to the last CItd.

### Limitations of the study

Despite inclusion of two independent collectives our data are derived from only two ICUs. Although our data suggest that the (in) accuracy of CIpc is predominantly associated to changes in CIpc compared to baseline CItd, we cannot definitely rule out a certain impact of time to last calibration due to the limited number of patients.

Regarding ethical considerations we did not extend the calibration-free observation-period above the maximum interval of 8 h suggested by the manufacturers.

At first glance the study design not including a pre-defined sequence of interventions (e.g. fluid-challenge, changes in vasoactive drugs) might be considered to be observational. However, based on clinical requirements most of the patients experienced substantial changes in treatment modalities during the 21 h observation-period including onset and termination of renal-replacement-therapy, changes in ventilator-settings and vasoactive drugs (Table 1).

## Conclusion

At present recalibration of CIpc by TPTD is suggested after a maximum of 8 h, although there is an ongoing debate to which extent accuracy of CIpc depends on the “time to last calibration”. By contrast, this study suggests that recalibration triggered by changes of the CIpc itself compared to the last calibration should be preferred to fixed intervals to last TPTD.

### Key messages

1. At present recalibration of CIpc by TPTD is suggested after a maximum of 8 h, although there is an ongoing debate to which extent accuracy of CIpc depends on the time to last calibration.
2. None of several analyses of this study supports that accuracy and/or precision of CIpc depend on the time to last calibration by TPTD.
3. By contrast, our data suggest that recalibration triggered by changes of CIpc itself compared to CItd(base) derived from the previous TPTD should be preferred to fixed intervals.
4. A “calibration-index” derived solely and continuously from pulse-contour-analysis might be a useful tool to improve the yield of relevant TPTD-measurements and to reduce “routine”-measurements after rigid intervals.
5. In addition to CIpc-CItd(base), changes in pulse pressure and/or dPmax might further improve a continuously derived “calibration-index” suggesting recalibration.

## Abbreviations

AUC: Area under the curve
AUSPC: Area under the systolic portion of the arterial pressure-curve
CO: Cardiac output
CI: Cardiac index
CIpc: Cardiac index derived from pulse contour analysis
CItd: Cardiac index derived from transpulmonary thermodilution
CItd(base): Cardiac index derived from the last (previous) transpulmonary thermodilution
CVC: Central venous catheter
dPmax: Index of left ventricular contractility
EVLWI: Extravascular lung-water index
GEDVI: Global end-diastolic volume index
GLMM: Generalized Linear Mixed Model
ICU: Intensive care unit
PCA: Pulse-Contour-Analysis
PE: Percentage error
PP: Pulse pressure
RRT: Renal replacement-therapy
ROC: Receiver operating characteristics
r_part_: Coefficient of correlation r derived from partial correlation analysis
TPTD: Transpulmonary thermodilution
